# Antipsychotic use and diagnosis of delirium in the intensive care unit

**DOI:** 10.1186/cc11342

**Published:** 2012-05-16

**Authors:** Joshua T Swan, Kalliopi Fitousis, Jeffrey B Hall, S Rob Todd, Krista L Turner

**Affiliations:** 1 College of Pharmacy and Health Sciences, Texas Southern University, Suite # 2-25G, 2450 Holcombe Blvd, Houston, TX 77004, USA; 2 The Methodist Hospital, 6565 Fannin St, DB1-09, Houston, TX 77030, USA; 3 New York University Langone Medical Center, 550 First Avenue, New Bellevue 15 East 9, New York, NY 10016, USA; 4 Surgical Intensive Care Unit, The Methodist Hospital, Department of Surgery, 6550 Fannin St., SM 1661A, Houston, TX 77030, USA; 5 Department of Surgery, Weill Cornell Medical College, 1300 York Avenue, New York, NY 10065, USA

## Abstract

**Introduction:**

Delirium is an independent risk factor for prolonged hospital length of stay (LOS) and increased mortality. Several antipsychotics have been studied for the treatment of intensive care unit (ICU) delirium that has led to a high variability in prescribing patterns for these medications. We hypothesize that in clinical practice the documentation of delirium is lower than the incidence of delirium reported in prospective clinical trials. The objective of this study was to document the incidence of delirium diagnosed in ICU patients and to describe the utilization of antipsychotics in the ICU.

**Methods:**

This was a retrospective, observational, cohort study conducted at 71 United States academic medical centers that reported data to the University Health System Consortium Clinical Database/Resource Manager. It included all patients 18 years of age and older admitted to the hospital between 1 January 2010 and 30 June 2010 with at least one day in the ICU.

**Results:**

Delirium was diagnosed in 6% (10,034 of 164,996) of hospitalizations with an ICU admission. Antipsychotics were administered to 11% (17,764 of 164,996) of patients. Of the antipsychotics studied, the most frequently used were haloperidol (62%; *n *= 10,958) and quetiapine (31%; *n *= 5,448). Delirium was associated with increased ICU LOS (5 vs. 3 days, *P *< 0.001) and hospital LOS (11 vs. 6 days, *P *< 0.001), but not in-hospital mortality (8% vs. 9%, *P *= 0.419). Antipsychotic exposure was associated with increased ICU LOS (8 vs. 3 days, *P *< 0.001), hospital LOS (14 vs. 5 days, *P *< 0.001) and mortality (12% vs. 8%, *P *< 0.001). Of patients with antipsychotic exposure in the ICU, absence of a documented mental disorder (32%, *n *= 5,760) was associated with increased ICU LOS (9 vs. 7 days, *P *< 0.001), hospital LOS (16 vs. 13 days, *P *< 0.001) and in-hospital mortality (19% vs. 9%, *P *< 0.001) compared to patients with a documented mental disorder (68%, *n *= 12,004).

**Conclusions:**

The incidence of documented delirium in ICU patients is lower than that documented in previous prospective studies with active screening. Antipsychotics are administered to 1 in every 10 ICU patients. When administration occurs in the absence of a documented mental disorder, antipsychotic use is associated with an even higher ICU and hospital LOS, as well as in-hospital mortality.

## Introduction

Delirium is an acute cognitive impairment defined by fluctuating mental status, inattention and disorganized thought. Intensive care unit (ICU) delirium is an independent risk factor for increased mortality, time on mechanical ventilation, ICU length of stay (LOS) and hospital LOS [1-4]. The Society of Critical Care Medicine recommends routine assessment in the ICU for the presence of delirium [5]. Despite the significant morbidity associated with delirium and this call for proactive screening, documentation of the disorder is highly variable. A recent survey of critical care health professionals found that 86% believe that delirium is underdiagnosed, 41% still do not screen for the disease and only 20% use a validated delirium-screening tool [6].

Several antipsychotics (haloperidol, olanzapine, quetiapine, risperidone and ziprasidone) have been studied for the treatment of ICU delirium [7-10]. Evidence-based guidelines for appropriate medical management of delirium are scant and a high variability of prescribing patterns has been observed [6]. Within our own institution, we have noted that some clinicians utilize the sedating properties of atypical antipsychotics in ICU patients to alleviate insomnia or treat acute agitation, but these patients may not have the diagnosis of delirium or a mental disorder. In other cases, some patients will be initiated on an antipsychotic in the ICU with little-to-no documentation regarding the indication for therapy. Because of these observations, we suspect that antipsychotics are currently used in the ICU when a clear indication for therapy is not specified. Delirium appears to be underappreciated and treated inappropriately with benzodiazepines or narcotics, which may exacerbate the delirious state in a dose-dependent relationship [1-3]. Prospective trials reveal the incidence of ICU delirium to be as high as 60% [3], with an even higher prevalence in the mechanically ventilated patient [1,2,11-14]. Data from our single institution with no active delirium screening found that International Classification of Diseases, ninth revision (ICD-9) codes for delirium were only documented in 2% of patients with an ICU admission and antipsychotics were administered to 9.8% of patients in the ICU [15]. Curiously, 43% of patients who were administered antipsychotics did not have an ICD-9 diagnosis code for a mental disorder or delirium. Based on these data, we suspect that in clinical practice the appreciation for and appropriate diagnosis of delirium are actually much lower than the incidence reported in trials with active delirium screening. Using a national database, we hypothesized that the incidence of documented delirium is low and that antipsychotic administration in the ICU occurs independently of a documented mental disorder (including delirium).

## Materials and methods

### Data source

We performed a retrospective cohort study using diagnosis and medication administration data submitted to the Clinical Database/Resource Manager (CDB/RM) at the University HealthSystem Consortium (UHC) [16]. The UHC is an alliance of 113 academic medical centers and 256 affiliated hospitals, which represent approximately 90% of the nation's non-profit academic medical centers. Consortium members enrolled in the database submit hospital encounter information upon patient discharge.

Among the information contained in the database are patient demographics, diagnosis and procedure codes, LOS, in-hospital mortality, ICU day resource utilization and medication administration in the ICU. The CDB/RM includes patient severity of illness and risk of mortality scores generated from 3M™ All Patient Refined Diagnosis Related Groups software (3M Health Information Systems, Salt Lake City, UT, USA) which uses ICD-9 diagnosis and procedure codes to classify patients into four categories each for severity of illness and risk of mortality: mild, moderate, major and extreme. Both admission and discharge scores are tabulated -- the former by excluding all diagnosis codes that were not present on admission, and the latter using all diagnosis codes submitted. UHC also generates flags for chronic/comorbid conditions based on definitions adopted by the Agency for Healthcare Research and Quality. The 29 comorbidities are correlated with resource utilization and severity of illness [17].

The UHC has created Medicare Severity Diagnosis Related Group level risk-adjustment models for three outcomes of care: cost, LOS and hospital mortality. When appropriate, the models incorporate admission severity of illness and risk of mortality, Agency for Healthcare Research and Quality chronic/comorbid conditions, age, gender, race, socioeconomic status (use payer source as a surrogate), admission status, admission source, and diagnoses and procedures pertinent to modeled patient populations. In-hospital mortality and LOS expected values are calculated for every discharge and can then be aggregated for various patient cohorts. The UHC CDB/RM reporting system generates aggregate results, and not patient specific output.

### Patient population

This study was approved by the Institutional Review Board of The Methodist Hospital Research Institute and was granted a waiver of informed consent. We included patient data submitted by all UHC-enrolled hospitals participating in the Clinical Resource Manager database (71 academic medical centers) with pharmacy clinical resources for all patients 18 years of age or older who were admitted from 1 January 2010 through 30 June 2010 with at least one day of ICU care. We used ICD-9 diagnosis codes to identify patients with delirium. Delirium was defined as the following ICD-9 diagnosis codes: 290.11, 290.3, 290.41, 291.0 to 291.9, 292.81, 293.0 to 293.1, 293.9, 300.11, 308.09, 780.02, and 780.09. Mental disorders, which include delirium, were defined as the following ICD-9 diagnosis codes: 290 to 319, 780.02, and 780.09. Exposure to antipsychotics was defined as medication utilization in the ICU and included the following medications: aripiprazole, clozapine, haloperidol oral, haloperidol decanoate injection, haloperidol lactate injection, olanzapine, paliperidone oral, paliperidone palmitate injection, quetiapine, risperidone and ziprasidone.

### Data elements

We reviewed patient characteristics (gender, race, sex, age over 65 years and admission severity of illness), diagnoses of mental disorders and delirium, antipsychotic exposure, and clinical outcomes (ICU LOS, hospital LOS, expected hospital LOS, in-hospital mortality and expected in-hospital mortality). Patient cohorts were compared based on documentation of delirium and antipsychotic exposure. A subgroup analysis of patients with antipsychotic exposure was performed comparing patients with a documented mental disorder versus those without.

### Statistical analysis

Descriptive statistics were generated from CDB/RM and displayed as percentages for nominal data and median accompanied by interquartile range (IQR) for continuous variables that were not normally distributed. Inferential statistics compared baseline characteristics, clinical outcomes and antipsychotic exposure between cohorts using chi-square tables and Fisher's exact tests for categorical variables and Mann-Whitney U tests for ordinal data and continuous variables that were not normally distributed. Odds ratio was calculated using binary logistic regression of two-by-two contingency tables. Data were tested for normality using the Anderson-Darling Normality Test with a *P*-value < 0.05 signifying nonparametric data. An α value of 0.05 was set for statistical significance.

## Results

### Baseline patient characteristics

**Figure 1:**
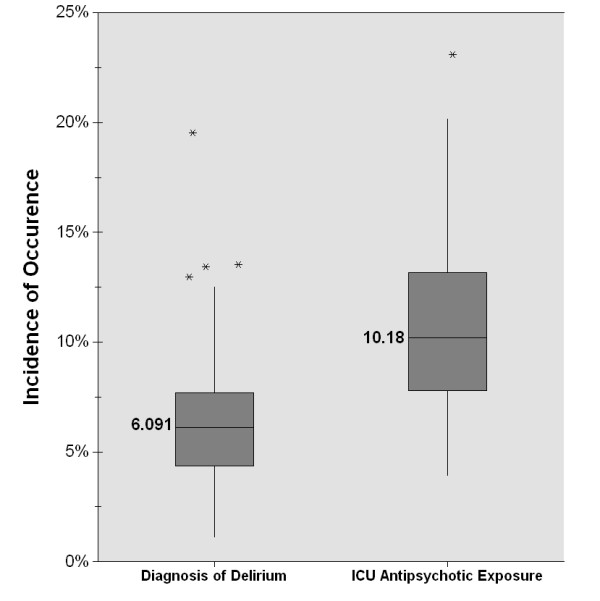
**Box plot showing incidence of documented delirium and antipsychotic exposure by hospital**. Results report data in aggregate by hospital for 71 Hospitals in the United States (*n *= 164,996). Medians labeled; Boxes, 25^th^, 50^th ^(median), and 75^th ^percentiles; bars, maximum and minimum values that were not outliers; asterisk, outliers; One extreme outlier (67%) for ICU Antipsychotic Exposure is not shown.

Between 1 January 2010 and 30 June 2010, there were 164,996 hospitalizations with at least one day of ICU stay. Fifty-six percent of patients were male, 64% were Caucasian, and 54% were admitted to a surgical ICU. Demographics for patients with regard to documentation of delirium and exposure to antipsychotics are summarized in Table 1. Documentation of delirium was present in only 6% (*n *= 10,034) of hospitalizations with an ICU admission. The incidence of documentation of delirium among individual hospitals was low (6%, IQR 4.3% to 7.7%), with no hospital reporting an incidence above 20% (Figure 1). The majority of delirium was documented as delirium due to a general medical condition (34%, *n *= 3,433; ICD-9 codes 293.0 to 293.1), alcohol withdrawal delirium (31%, *n *= 3,060; ICD-9 codes 291 to 291.9), and delirium not otherwise specified (27%, *n *= 2,698; ICD-9 code 780.09). Documentation of a mental disorder, including delirium, was present in 43% (*n *= 70,206) of hospitalizations with an ICU admission.

**Table 1:** Basic demographics and clinical characteristics of patients

Demographics and clinical characteristics	All patients 164,996 (100%)	Delirium 10,034 (6.1%)	No delirium 154,962 (93.9%)	*P*-value	Antipsychotic exposure 17,764 (10.6%)	No antipsychotic exposure 140,865 (85.4%)*^a^*	*P*-value
Age ≥65 years, n (%)	59,550	3,937	55,613	<	6,444	50,383	0.181
	(36.1)	(39.3)	(35.7)	0.001	(36.3)	(35.8)	
Male sex, n (%)	92,157	6,499	85,658	<	10,884	77,686	< 0.001
	(55.9)	(64.8)	(55.3)	0.001	(61.3)	(55.2)	
Surgical ICU type, n (%)	89,423	4,850	84,573	<	9,930	76,693	< 0.001
	(54.2)	(48.3)	(54.6)	0.001	(55.9)	(54.4)	
Race							
White, n (%)	105,565	7,072	98,493	<	12,272	89,055	< 0.001
	(64.0)	(70.5)	(63.6)	0.001	(69.1)	(63.2)	
Black, n (%)	34,647	1,647	33,000	<	3,173	30,061	< 0.001
	(21.0)	(16.4)	(21.3)	0.001	(17.9)	(21.3)	
Other, n (%)	24,784	1,315	23,469	<	2,319	21,749	< 0.001
	(15.0)	(13.1)	(15.1)	0.001	(13.1)	(15.4)	
Admit severity of illness							
Minor, n (%)	23,619	526	23,093	<	993	18,007	< 0.001
	(14.3)	(5.2)	(14.9)	0.001	(5.6)	(14.7)	
Moderate, n (%)	47,543	2,064	45,479	<	3,150	35,956	< 0.001
	(28.8)	(20.6)	(29.3)	0.001	(17.7)	(29.4)	
Major, n (%)	57,747	4,067	53,680	<	6,386	42,471	0.002
	(35.0)	(40.5)	(34.6)	0.001	(36.0)	(34.7)	
Extreme, n (%)	36,083	3,377	32,706	<	7,234	25,830	< 0.001
	(21.9)	(33.7)	(21.1)	0.001	(40.7)	(21.1)	

Results compare patients with an admission to an intensive care unit with documentation of delirium versus none and with antipsychotic exposure versus none (*n *= 164,996). *^a^*Due to equivocal data for antipsychotic administration, 4% (*n *= 6,337) of patients were excluded from antipsychotic exposure versus no antipsychotic exposure comparisons. It is likely that these patients received an antipsychotic while hospitalized, but administration while in the ICU could not be confirmed. ICU, intensive care unit

**Figure 2:**
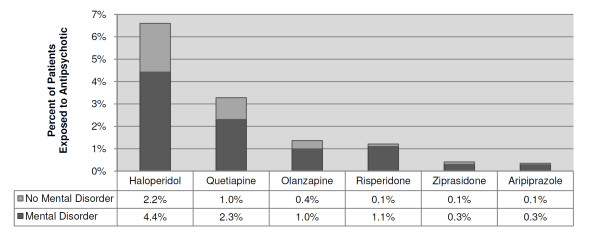
**Individual antipsychotic frequency of use**. Results show data for patients with an admission to an intensive care unit with and without documentation of a mental disorder (*n *= 164,996). Data are not shown for antipsychotics which were used in less than 1% of admissions: clozapine (0.4%) and paliperidone (0.2%).

Exposure to antipsychotics in the ICU occurred in 11% (*n *= 17,764) of hospitalizations with an ICU admission. The incidence of antipsychotic exposure among individual hospitals was variable (10%, IQR 7.8% to 13.2%) (Figure 1). Of the antipsychotics studied, the most frequently used was haloperidol (62%; *n *= 10,958) followed by quetiapine (31%; *n *= 5,448), risperidone (15%; *n *= 2,692), olanzapine (13%; *n *= 2,262), ziprasidone (4%; *n *= 684), aripiprazole (3%; *n *= 584), clozapine (0.4%; *n *= 71), and paliperidone (0.2%; *n *= 40) (Figure 2). Patients with documentation of delirium were more likely to receive an antipsychotic versus patients without documentation of delirium (39% vs. 9%, *P *< 0.001) (Table 2). This study included three atypical antipsychotics (aripiprazole, clozapine and paliperidone) that have not been studied for the treatment of delirium. These three agents were rarely used (0.4%, 695 of 164,996), given predominately to patients with a documented mental disorder (84%, 583 of 695), and given infrequently to patients with documented delirium (12%, 86 of 695). Only 68% (12,004 of 17,764) of patients with antipsychotic exposure had documentation of a mental disorder (Table 3).

**Table 2:** Individual antipsychotic frequency of use

	Delirium 10,034 (6.1%) , n (%)	No delirium 154,962 (93.9%) , n (%)	**Odds ratio**, (95% CI)	*P*-value
Antipsychotic exposure	3,883	13,880	6.4 (6.1 to 6.7)	< 0.001
	(38.7)	(9.0)		
Haloperidol	3,014	7,969	7.9 (7.6 to 8.3)	< 0.001
	(30.0)	(5.1)		
Quetiapine	1,271	4,189	5.2 (4.9 to 5.6)	< 0.001
	(12.7)	(2.7)		
Risperidone	497	2,199	5.8 (5.2 to 6.3)	< 0.001
	(5.0)	(1.4)		
Olanzapine	592	1,676	3.6 (3.3 to 4.0)	< 0.001
	(5.9)	(1.1)		
Ziprasidone	140	545	4.0 (3.3 to 4.8)	< 0.001
	(1.4)	(0.4)		
Aripiprazole	75	509	2.3 (1.8 to 2.9)	< 0.001
	(0.7)	(0.3)		
Clozapine	10	61	2.5 (1.3 to 5.0)	< 0.001
	(0.1)	(0.0)		
Paliperidone	1	39	0.4 (0.1 to 2.9)	0.516
	(0.0)	(0.0)		

Results show data for patients with an admission to an intensive care unit with and without documentation of a mental disorder (*n *= 164,996). CI, confidence interval

**Table 3:** Basic demographics and clinical characteristics of patients with antipsychotic exposure

Demographics and clinical characteristics	Antipsychotic exposure and mental disorder 12,004 (68%)	Antipsychotic exposure and no mental disorder 5,760 (32%)	*P*-value
Age ≥65 years, n (%)	3,807	2,637	< 0.001
	(31.7)	(45.8)	
Male sex, n (%)	7,293	3,591	0.042
	(60.8)	(62.4)	
Surgical ICU type, n (%)	6.177	3.753	< 0.001
	(51.5)	(65.2)	
Race			
White, n (%)	8,359	3,913	0.021
	(69.6)	(67.9)	
Black, n (%)	2,192	981	0.045
	(18.3)	(17.0)	
Other, n (%)	1,453	866	< 0.001
	(12.1)	(15.0)	
Admit severity of illness			
Minor, n (%)	694	299	0.109
	(5.8)	(5.2)	
Moderate, n (%)	2,287	864	< 0.001
	(19.1)	(15.0)	
Major, n (%)	4,434	1,951	< 0.001
	(36.9)	(33.9)	
Extreme, n (%)	4,588	2,646	< 0.001
	(38.2)	(45.9)	

Results show data for patients with an admission to an ICU with administration of an antipsychotic while in the ICU (*n *= 17,764). Table compares data for patients with and without documentation of a mental disorder. ICU, intensive care unit

### Clinical outcomes

For all hospitalizations with an ICU admission, the median ICU LOS was three days (IQR 2 to 5), median hospital LOS was six days (IQR 3 to 12), and in-hospital mortality was 9%. Patients with documentation of delirium versus patients without documentation of delirium had an increased ICU LOS (5 vs. 3 days, *P *< 0.001) and hospital LOS (11 vs. 6 days, *P *< 0.001), but not in-hospital mortality (8% vs. 9%, *P *= 0.419). Based on UHC CDB/RM hospital LOS and mortality model predictions, patients with delirium stayed in the hospital longer than expected (11 observed days vs. 10 expected days, *P *< 0.001) but die in-hospital less often than expected (8% observed mortality vs. 12% expected mortality, *P *< 0.001). Outcomes for patients with regard to documented delirium are summarized in Table 4.

**Table 4:** Clinical outcomes of patients

Clinical outcomes	Delirium 10,034 (6.1%)	No delirium 154,962 (93.9%)	*P-value*	Antipsychotic exposure 17,764 (10.6%)	No antipsychotic exposure 140,865 (85.4%)*^a^*	*P*-value
ICU LOS, median days (IQR)	5 (3 to 10)	3 (2 to 5)	< 0.001	8 (4 to 15)	3 (2 to 4)	< 0.001
Hospital LOS						
Observed, median days (IQR)	11 (6 to 19)	6 (3 to 11)	< 0.001	14 (7 to 24)	5 (3 to 10)	< 0.001
Expected, median days (IQR)	10 (7 to 15)	7 (5 to 10)	< 0.001	13 (9 to 21)	7 (5 to 10)	< 0.001
Mortality						
Observed, %	8.3	8.6	0.419	12.2	8.1	< 0.001
Expected, %	11.5	8.3	< 0.001	14.0	7.8	< 0.001

Results compare outcomes for patients with an admission to an intensive care unit with documentation of delirium versus none and with antipsychotic exposure versus none (*n *= 164,996). *^a^*Due to equivocal data for antipsychotic administration, 4% (*n *= 6,367) of patients were excluded from antipsychotic exposure versus no antipsychotic exposure comparisons. ICU, intensive care unit; LOS, length of stay

Patients exposed to antipsychotics in the ICU versus patients without antipsychotic exposure had an increased ICU LOS (8 vs. 3 days, *P *< 0.001), hospital LOS (14 vs. 5 days, *P *< 0.001), and in-hospital mortality (12% vs. 8%, *P *< 0.001). Based on UHC CDB/RM hospital LOS and mortality model predictions, patients with antipsychotic exposure stayed in the hospital longer than expected (14 observed days vs. 13 expected days, *P *< 0.001) but die in-hospital less often than expected (12% observed mortality vs. 14% expected mortality, *P *< 0.001). Outcomes for patients with regard to antipsychotic exposure are summarized in Table 4.

Subgroup analysis of patients with antipsychotic exposure found patients without a documented mental disorder diagnosis, as compared to patients with a mental disorder, had an increased ICU LOS (9 vs. 7 days, *P *< 0.001), hospital LOS (16 vs. 13 days, *P *< 0.001), and in-hospital mortality (19% vs. 9%, *P *< 0.001) (Table 5). Based on UHC CDB/RM hospital LOS and mortality prediction models, patients exposed to antipsychotics without a documented mental disorder stayed in the hospital longer than expected (16 observed days vs. 14 expected days; *P *< 0.001) and died in-hospital more often than expected (19% observed mortality vs. 16% expected mortality; *P *< 0.001).

**Table 5:** Clinical outcomes of patients with antipsychotic exposure

Clinical outcomes	Antipsychotic exposure and mental disorder 12,004 (68%)	Antipsychotic exposure and no mental disorder 5,760 (32%)	*P*-value
ICU LOS, median days (IQR)	7 (4 to 14)	9 (4 to 18)	< 0.001
Hospital LOS			
Observed, median days (IQR)	13 (7 to 23)	16 (8 to 28)	< 0.001
Expected, median days (IQR)	12 (9 to 20)	14 (9 to 22)	< 0.001
Mortality			
Observed, %	9.0	18.7	< 0.001
Expected, %	12.8	16.4	< 0.001

Results show data for patients with an admission to an ICU with administration of an antipsychotic while in the ICU (*n *= 17,764). Table compares data for patients with and without documentation of a mental disorder. ICU, intensive care unit. ICU, intensive care unit; IQR, interquartile range; LOS, length of stay.

## Discussion

Despite the fact that prospective clinical trials with active screening for delirium have shown that delirium occurs in 20% to 60% of critically ill patients [1-3,11-14], this retrospective review of 71 academic medical centers in the United States found that this disease is only documented in 6% of critically ill patients. Therefore, a majority of delirium is either undiagnosed or the diagnosis of delirium is not being documented using ICD-9 diagnosis codes. Contemporary clinical trials demonstrate that delirium is an independent risk factor of mortality and other unfavorable outcomes [1-4], and the absence of recognition of delirium using ICD-9 diagnosis codes represents an opportunity for improvement for the critical care medicine community.

The low incidence of documented delirium has led the authors to hypothesize that most hospitals (similar to our institution) were not actively screening for delirium [6] as recommended in the 2002 Society of Critical Care Medicine guidelines [5]. In the absence of active screening, it is likely that only cases of the highly-visible hyperactive type of delirium (a minority subset of delirium) were diagnosed and documented with ICD-9 diagnosis codes and the less-visible hypoactive type of delirium (the majority of delirium) was missed [1]. Even though physicians and nurses may feel confident in their ability to diagnose delirium, using clinical judgment for diagnosis of delirium misses 70% of delirium compared to a standardized screening tool such as the Confusion Assessment Method for the ICU (CAM-ICU) [18]. The CAM-ICU is a validated delirium screening tool that takes an average of two minutes to conduct and can be implemented with minimal training [11,13]. Institutions that are interested in adopting the CAM-ICU screening tool can obtain free access to training manuals, reference material and training videos online at the ICU Delirium and Cognitive Impairment Study Group website [19].

This study found that the documentation of delirium is associated with a significant increase in ICU and hospital LOS, but not mortality. The clinician should use caution when interpreting this outcome data, as the 6% incidence of documented delirium was much less than the incidence of 20% to 60% [1-3,11-14] in studies with prospectively collected data with routine delirium-screening assessments. Expectedly, patients with documented delirium had a higher severity of illness at admission, which is likely to increase LOS. Severity of illness is a variable used to generate the UHC expected LOS. In this study, the observed LOS was longer than the expected LOS for patients with documented delirium. This study is unable to determine if delirium causes an increase in the ICU LOS and hospital LOS or if patients with increased ICU LOS and hospital LOS are more likely to be diagnosed or receive documentation of a diagnosis. Patients with documented delirium had similar in-hospital mortality compared to patients without documented delirium, even though several prospective observational studies have correlated delirium with a significant increase in in-hospital mortality [2,4]. Also, the incidence of observed mortality was less than the UHC-expected mortality for these patients with documented delirium. The authors hypothesize that delirium was not associated with mortality in this study because only a small subset (most likely the hyperactive type) of truly delirious patients received a documentation of delirium using ICD-9 diagnosis codes.

Antipsychotics were administered in the ICU to 11% of hospitalizations with an ICU admission. Haloperidol was the most frequently used antipsychotic, which is consistent with the 2002 Society of Critical Care Medicine guidelines [5] and a recent survey [6] in which 86% of health professionals reported using haloperidol to treat delirium compared to 40% who reported using atypical antipsychotics. This study found that patients with documented delirium were six times more likely to receive an antipsychotic. Since the publication of the 2002 Society of Critical Care Medicine guidelines, trials have studied four atypical antipsychotics (quetiapine, olanzapine, risperidone and ziprasidone) for the treatment of ICU delirium [7-10]. These trials have consisted of very small sample sizes and must be interpreted with caution. Of these four atypical antipsychotics, these results found quetiapine to be used most often in ICU patients. Ziprasidone was used least often, which may reflect hesitation to use this medication since it carries a black box warning for QTc prolongation. Exposure to antipsychotics was associated with increased ICU LOS, hospital LOS and in-hospital mortality. However, the observed mortality was less than the expected mortality for these patients. It is unknown if exposure to antipsychotics caused increased ICU LOS and hospital LOS, or if patients with longer hospitalizations were more likely to be exposed to an antipsychotic because they stayed longer. The correlation between antipsychotics and outcomes in this retrospective study may be heavily influenced by selection bias, and patients who were refractory to other interventions may have been more likely to receive antipsychotic therapy. Large prospective randomized trials are needed to determine if there is a cause-and-effect relationship between antipsychotic exposure in the ICU and ICU LOS, hospital LOS and in-hospital mortality.

A subgroup analysis of patients who were exposed to antipsychotics was conducted to elicit any potential differences in patient demographics and clinical outcomes for patients regarding documentation of a mental disorder (which served as a surrogate marker for indication of therapy). Of patients exposed to antipsychotics, 32% did not have documentation of a mental disorder. Patients exposed to antipsychotics without documentation of a mental disorder were also more likely to be elderly and have an extreme admission severity of illness, which is concerning, as atypical antipsychotics carry a black box warning for increased mortality in elderly patients. Clinical outcomes were different between groups as patients without documentation of a mental disorder had increased ICU LOS, hospital LOS and over twice the mortality compared to patients with documentation of a mental disorder. This study found that neither the documentation of delirium nor the exposure to antipsychotic medication in the ICU alone were associated with an in-hospital mortality that was higher than expected. However, observed morality was higher than expected for patients who received antipsychotics when there was no documentation of a mental disorder (including delirium).

Several limitations must be considered when interpreting this study. This observational study found an unusually low incidence of documented delirium that is mostly likely attributable to low rates of documentation of diagnosed delirium or a low incidence of delirium diagnosis due to improper screening. This study utilized ICD-9 diagnosis codes that are collected for billing purposes and not for research. The low incidence of documented delirium found in this trial should not be misconstrued to indicate a low rate of disease occurrence, since the methods used to screen for delirium at these hospitals is unknown. Low rates of documentation could be explained by variable nomenclature (that is, acute encephalopathy, ICU psychosis, toxic psychosis, and so on) that may result in inaccurate ICD-9 diagnosis coding. The authors tried to account for this by using 11 ICD-9 codes for delirium. The authors are unable to estimate how many patients were diagnosed with delirium, yet never received documentation of this diagnosis using one of these 11 ICD-9 codes. The UHC database allows up to 99 ICD-9 codes per admission, and it is unlikely that ICD-9 codes reported from the hospital were truncated in the UHC database. One hypothesized limitation was that ICD-9 codes were only being collected for reimbursement (MS-DRG accounts for the first nine ICD-9 codes). Further review of the UHC database found that at least 40% of patients had more than nine ICD-9 codes and 50% of the ICD-9 codes for delirium occurred after the ninth position. Based on this information, the authors believe that the potential impact of hospitals reporting ICD-9 codes to UHC that are only associated with financial incentive is minimal. This study is unable to determine what diagnostic tool, if any, was used for the diagnosis of delirium. This UHC CDB/RM electronic data are reported in groups by hospital or in aggregate; therefore, we were unable to perform multivariate analysis to correlate delirium or antipsychotic exposure as an independent risk factor for clinical outcomes. Patient variables, such as biomarkers of organ function, were not available in this database. Therefore, this study was unable to compare patient groups using traditional baseline severity of illness scoring systems. However, UHC has provided admission severity of illness, expected hospital LOS and expected mortality for patients using reggression-modeling and independent risk factors. We have compared observed data to these expected values where applicable. Diagnosis codes are recorded for each hospitalization and are not specific for diagnosis during ICU days. This study did not capture the incidence of ICD-9 codes that were present-on-admission versus new diagnoses.

There were equivocal data regarding antipsychotic medication use for 4% (*n *= 6,367) of patients. It is likely that these patients received an antipsychotic while hospitalized, but administration while in the ICU could not be confirmed. Therefore, these patients were excluded from antipsychotic exposure versus no antipsychotic exposure comparisons. We were not able to quantify the dose or number of doses of antipsychotic administered to patients, and data were analyzed dichotomously as "exposed" or "not exposed." While several formulations of haloperidol were available as options in the database, a sub analysis of intravenous versus oral haloperidol was not conducted since many hospitals did not specify the formulation that was used. Regarding the subgroup analysis of patients receiving antipsychotics, this study is unable to determine the clinician's intended indication for antipsychotic therapy. All approved indications for antipsychotic therapy were accounted for in the group of ICD-9 diagnosis codes used to define a mental disorder.

## Conclusions

This report demonstrates that the incidence of documented delirium in ICU patients is lower than that documented in previous prospective studies. We hypothesize that the low rates of documented delirium using ICD-9 diagnostic codes is attributable to improper screening of delirium, which was the case at our institution. This study suggests that hospitals are not routinely assessing for delirium as recommended by the Society of Critical Care Medicine and that a national culture change is needed to increase the awareness and diagnosis of delirium.

Antipsychotics are administered to 1 in every 10 ICU patients, and exposure to these medications is associated with increased ICU and hospital LOS. Patients exposed to an antipsychotic, when there is no documentation of a mental disorder, have increased ICU LOS, hospital LOS and mortality compared to patients with documentation of a mental disorder. These findings do not support the use of antipsychotic medications in the ICU when patients do not have a documented diagnosis of a mental disorder or delirium. The appropriate indication and agent selection of the antipsychotics should continue to be studied in prospective, randomized, controlled trials. Due to the high prevalence of antipsychotic use in ICU patients who do not have a documented mental disorder, future studies are needed to describe the specific indications for antipsychotics and common doses that are being used in critically ill patients in current clinical practice.

## Key messages

• The incidence of documented delirium in a nationwide cohort was only 6% in patients who had at least one day of ICU care, which is likely attributable to poor rates of screening, diagnosis and documentation.

• Antipsychotic medications were administered in the ICU to 11% of patients.

• Patients with documented delirium were six times more likely to receive antipsychotics in the ICU.

• The most frequently used antipsychotic was haloperidol.

• Patients exposed to antipsychotic medications when there is no documented diagnosis of a mental disorder (including delirium) had worse clinical outcomes.

• These findings do not support the use of antipsychotic medications in the ICU when patients do not have a documented diagnosis of a mental disorder or delirium.

## Abbreviations

CDB/RM: Clinical Database/Resource Manager; CI: confidence interval; ICD-9: International Classification of Diseases: ninth revision; ICU: intensive care unit; IQR: interquartile range; LOS: length of stay; UHC: University HealthSystem Consortium.

## Competing interests

This was unfunded research. All authors declare that they have no competing interests.

## Authors' contributions

JTS was the principal investigator and conceived the original study hypothesis and design. JTS, KF, JBH, SRT and KLT contributed to the study conception and design, participated in data analysis and drafted the manuscript. All authors helped revise the manuscript. All authors read and approved the final manuscript.

## Authors' information

When this research was initiated, JTS was a PGY2 Critical Care Medicine Pharmacy Resident at The Methodist Hospital. JTS is currently an Assistant Professor of Pharmacy Practice at Texas Southern University, where this manuscript was completed. KF and JBH are Critical Care Clinical Pharmacist Specialists at The Methodist Hospital and served as research mentors for the PGY2 Critical Care Medicine Pharmacy Residency. When this research was initiated, SRT and KLT were both critical care physicians and surgeons at The Methodist Hospital. KLT currently serves as the Medical Director for the Surgical ICU at The Methodist Hospital.
